# CD39+CD55− Fb Subset Exhibits Myofibroblast-Like Phenotype and Is Associated with Pain in Osteoarthritis of the Knee

**DOI:** 10.3390/biomedicines11113047

**Published:** 2023-11-14

**Authors:** Maho Tsuchiya, Yoshihisa Ohashi, Yoshio Kodera, Masashi Satoh, Takashi Matsui, Kensuke Fukushima, Dai Iwase, Jun Aikawa, Manabu Mukai, Gen Inoue, Masashi Takaso, Kentaro Uchida

**Affiliations:** 1 Department of Orthopaedic Surgery, Kitasato University School of Medicine, 1-15-1 Minami-ku, Kitasato, Sagamihara 252-0374, Kanagawa, Japan; 09.ma.10.ho@gmail.com (M.T.); 44134413oo@gmail.com (Y.O.); kenfu@r4.dion.ne.jp (K.F.); daiiwase19760601@yahoo.co.jp (D.I.); jun43814@gmail.com (J.A.); m.manabu0829@hotmail.co.jp (M.M.); ginoue@kitasato-u.ac.jp (G.I.); mtakaso@kitasato-u.ac.jp (M.T.); 2 Department of Physics, School of Science, Kitasato University, 1-15-1 Kitasato, Minami-ku, Sagamihara 252-0373, Kanagawa, Japan; kodera@kitasato-u.ac.jp (Y.K.); matsui@kitasato-u.ac.jp (T.M.); 3 Center for Disease Proteomics, School of Science, Kitasato University, 1-15-1 Kitasato, Minami-ku, Sagamihara 252-0373, Kanagawa, Japan; 4 Department of Immunology, Kitasato University School of Medicine, 1-15-1 Minami-ku, Kitasato, Sagamihara 252-0374, Kanagawa, Japan; msato@med.kitasato-u.ac.jp

**Keywords:** knee osteoarthritis, fibroblast subset, CD39, pain

## Abstract

Recent studies utilizing single-cell analysis have unveiled the presence of various fibroblast (Fb) subsets within the synovium under inflammatory conditions in osteoarthritis (OA), distinguishing them from those in rheumatoid arthritis (RA). Moreover, it has been reported that pain in knee OA patients is linked to specific fibroblast subsets. Single-cell expression profiling methods offer an incredibly detailed view of the molecular states of individual cells. However, one limitation of these methods is that they require the destruction of cells during the analysis process, rendering it impossible to directly assess cell function. In our study, we employ flow cytometric analysis, utilizing cell surface markers CD39 and CD55, in an attempt to isolate fibroblast subsets and investigate their relationship with OA pathology. Synovial tissues were obtained from 25 knee OA (KOA) patients. Of these, six samples were analyzed by RNA-seq (*n* = 3) and LC/MS analysis (*n* = 3). All 25 samples were analyzed to estimate the proportion of Fb (CD45−CD31−CD90+) subset by flow cytometry. The proportion of Fb subsets (CD39+CD55− and CD39−CD55+) and their association with osteoarthritis pathology were evaluated. CD39+CD55− Fb highly expressed myogenic markers such as CNN1, IGFBP7, MYH11, and TPM1 compared to CD39−CD55+ Fb. Kyoto Encyclopedia of Genes and Genomes (KEGG) analysis of upregulated differentially expressed genes (DEGs) in CD39+CD55− Fb identified the Apelin pathway and cGMP-PKC-signaling pathway as possibly contributing to pain. LC/MS analysis indicated that proteins encoded by myogenic marker genes, including CNN1, IGFBP7, and MYH11, were also significantly higher than in CD39−CD55+ Fb. CD39−CD55+ Fb highly expressed PRG4 genes and proteins. Upregulated DEGs were enriched for pathways associated with proinflammatory states (‘RA’, ‘TNF signaling pathway’, ‘IL-17 signaling pathway’). The proportion of CD39+CD55− Fb in synovium significantly correlated with both resting and active pain levels in knee OA (KOA) patients (resting pain, ρ = 0.513, *p* = 0.009; active pain, ρ = 0.483, *p* = 0.015). There was no correlation between joint space width (JSW) and the proportion of CD39+CD55− Fb. In contrast, there was no correlation between the proportion of CD39−CD55+ Fb and resting pain, active pain, or JSW. In conclusion, CD39+CD55− cells exhibit a myofibroblast phenotype, and its proportion is associated with KOA pain. Our study sheds light on the potential significance of CD39+CD55− synovial fibroblasts in osteoarthritis, their myofibroblast-like phenotype, and their association with joint pain. These findings provide a foundation for further research into the mechanisms underlying fibrosis, the impact of altered gene expression on osteoarthritic joints, and potential therapeutic strategies.

## 1. Introduction

Osteoarthritis (OA) is a prevalent and debilitating joint disorder that poses a significant global health burden. It is characterized by the gradual deterioration of joint tissues, leading to pain, stiffness, and impaired joint function. OA is not limited to the articular cartilage but affects the entire joint structure, including the cartilage, meniscus, subchondral bone, infrapatellar fat pad, and synovial membrane.

The synovial membrane experiences a variety of structural changes during the pathogenesis of osteoarthritis (OA). Synovium is composed predominantly of two cell types: macrophage-like and fibroblast-like cells (Fb) [1]. To date, many studies have reported the presence of heterogeneous macrophages such as M1 and M2 macrophages in synovium and noted that they contribute to osteoarthritis pathology [2,3,4]. Interestingly, in the early stages of the disease, synovitis becomes apparent even before significant radiographic evidence of cartilage damage. During this phase, synovial fibroblasts proliferate, serving as a primary source of the elevated pro-inflammatory cytokines detected in osteoarthritic synovial joints [5,6].

Recent investigations, employing spatial and single-cell analysis techniques, have unveiled the existence of distinct fibroblast subsets within the synovium under inflammatory conditions in osteoarthritis (OA) [7,8]. These subsets not only differentiate OA from rheumatoid arthritis (RA) but have also been associated with pain in knee OA patients [7,8]. While single-cell expression profiling methods offer a detailed insight into the molecular states of individual cells, it is important to note that they come with a limitation—they require cell destruction during the analysis process, making it impossible to assess cell function. As a result, the identification of cell surface markers specific to pain-related fibroblasts becomes a crucial step in characterizing these fibroblasts. However, it is worth mentioning that specific cell surface markers for pain-related fibroblasts have yet to be identified.

Various surface markers have been used to identify Fb subsets in tumor environments, scars, and RA, such as CD26, CD39, CD55, and FAP [9,10,11,12,13,14]. CD55, a glycosylphosphatidylinositol anchor protein, is expressed by Fb with high local abundance in the intimal lining layer [15]. CD39 converts ATP (or ADP) to adenosine monophosphate (AMP), which is converted into adenosine by CD73 [16]. CD39 expression has been found in mesenchymal stem cell-like cells derived from synovium [17]. However, a previous study reported that the CD39+ Fb subset contributes to fibrogenesis in skin [10]. During the entire course of fibrogenesis, the synovium undergoes hypertrophy and the development of fibrotic masses, which contribute to the chronic joint pain and stiffness experienced by OA [18,19,20]. We hypothesized that the CD39+ Fb subset contributes to OA pathology.

Here, to enhance the characterization of these fibroblast (Fb) subsets, we isolated specific Fb subsets based on cell surface markers through cell sorting and conducted a comprehensive investigation into the relationship between synovial Fb subsets, osteoarthritis (OA) pathology, and transcriptome and proteomics analysis.

## 2. Materials and Methods

### 2.1. Patients and Methods

Twenty-five patients diagnosed with knee osteoarthritis (KOA) in the Kellgren–Lawrence (KL) grades 3–4 who underwent total knee arthroplasty (TKA) were included in this study. Patients with a history of autoimmune diseases, such as rheumatoid arthritis (RA), were excluded from the study to minimize potential confounding factors. Patients with a history of significant joint trauma or surgery on the knee joint were excluded. The study protocol was approved by the Ethics Review Board of our institution (approval number: KMEO (B22–044)), and the study complied with the principles of the Declaration of Helsinki. All subjects provided written informed consent for both participation and the excision of synovial tissue before TKA. All participants underwent TKA at our institution, with sampling of the synovial membrane (SM).

### 2.2. Flow Cytometric Analysis and Cell Sorting

To identify the Fb subset in OA synovium and its phenotype, flow cytometric analysis and cell sorting were performed (*n* = 25). Each SM sample was digested with a 2 mg/mL type I collagenase solution for 2 h at 37 °C in a shaking incubator. After collagenase digestion, synovial cells isolated from each patient were subsequently reacted with Brilliant violet 421-conjugated anti-human CD31 (endothelial maker), APC/Cy7-conjugated CD39 (BioLegend, San Diego, CA, USA), FITC-conjugated anti-human CD45 (leucocyte marker, BioLegend), PE-conjugated anti-human CD55 (BioLgend), and PE-Cy7-conjugated anti-human CD90 (pan fibroblast marker) (Supplementary Table S1). The synovial fibroblasts from each patient were sorted individually. After two washes in PBS, cells were analyzed using a cell sorter (MA900, SONY, Tokyo, Japan). No staining control and single-stained samples were used to determine the negative gate for CD31, CD45, and the positive gate for CD90 (Supplementary Figure S1A–J). Cells that were treated with anti-CD31, CD45, and CD90 antibodies but not with anti-CD39 and CD55 antibodies were employed to determine the positive gate for CD39 and CD55 in the Fb population (Supplementary Figure S2A–F). The correlation proportions of Fb subsets (CD39−CD55+ and CD39+CD55−), the visual analog scale (VAS), and the joint space width (JSW) were then estimated. CD39−CD55+ Fb and CD39+CD55− Fb from 6 samples were also sorted and used to perform transcriptome analysis (RNA-Seq analysis, *n* = 3, LC-MS, *n* = 3).

### 2.3. Transcriptome Analysis

To identify genes that exhibited differential expression between CD39−CD55+ and CD39+CD55− Fb from three patients, total RNA was extracted using Trizol (Thermo Scientific, Waltham, MA, USA) and a spin column (Direct-zolbiol MicroPrep kit, Zymo Research, Orange, CA, USA). RNA quantity was determined using a spectrophotometer (Denovix, Wilmington, DE, USA), and RNA quality was assessed with an Agilent 2100 BioAnalyzer (Agilent Technologies, Santa Clara, CA, USA) utilizing an RNA 6000 Nano Chip. Subsequently, RNA-seq analysis was performed on the isolated RNA using DNBSEQ (DNBSEQ Technology Platform, BGI, Shenzhen, China) before the RNA samples were sent to BGI Japan for further RNA-seq analysis. Differential gene expression was identified using specific criteria in the RNA-seq analysis, wherein genes with a false discovery rate (FDR) ≤ 0.001 and a log2 fold change ≥1 were considered significant [21]. Pathway analysis utilized the Kyoto Encyclopedia of Genes and Genomes (KEGG; http://www.genome.jp/kegg/, accessed on 14 April 2023) for exploration of significant pathways. Key driver gene analysis (KDA) was conducted using the BGI online system (Dr.Tom). In detail, KDA analysis takes as input a set of genes (G) and a directed gene network (N) and aims to identify those key regulators of the gene set associated with a particular network [22,23].

### 2.4. LC-MS/MS Analysis

To identify proteins that were differentially expressed between CD39−CD55+ and CD39+CD55− Fb, cells obtained from 3 patients were analyzed by LC-MS. Sorted cells were centrifuged and washed three times with PBS, and the cell pellets were disrupted using a Bioruptor sonicator (SONIC Bio Co., Kanagawa, Japan), employing a high setting for 30 min with intermittent 30 s cycles on and off. The process is carried out in an ice water bath, and 20 µL of a phase-transfer surfactant (PTS) containing 12 mM sodium deoxycholate, 12 mM sodium lauryl sulfate, and 200 mM triethylammonium bicarbonate (TEAB) is added for each batch of ≤10^5^ cells. The primary purpose of this step is to break down cell membranes and facilitate protein extraction. Following sonication, the mixture undergoes centrifugation at 19,000× *g* for 15 min at 4 °C. This centrifugation step is essential for separating insoluble components from the supernatant. Disulfide bonds within the sample are reduced by incubating the supernatant with a 200 mM Bond-Breaker TCEP solution (Thermo Fisher Scientific, Waltham, MA, USA). This incubation occurs for 30 min at 50 °C, followed by an additional 10 min incubation on ice. Then, 375 mM iodoacetamide and 200 mM TEAB are introduced into the sample to alkylate the reduced cysteine residues. The incubation takes place in the dark at room temperature for 30 min and is a crucial step to prevent the reformation of disulfide bonds. The sample undergoes proteolytic digestion by 2 μL Lys-C (0.1 mg/mL) and 2 μL trypsin (0.1 mg/mL) at 37 °C for 18 h. This process breaks down proteins into peptides, which can be further subjected to analysis. To remove PTS from the digest, a 1.5× volume of a solution comprising 1.7% trifluoroacetic acid (TFA) is added. The digest is then centrifuged at 19,000× *g* at 4 °C for 15 min. The supernatant is desalted, and peptides are extracted using StageTips equipped with a C18 Empore disk membrane, as described elsewhere [24]. The peptides are eluted by employing a solution of 0.1% TFA and 50% acetonitrile (ACN), after which they are freeze-dried to concentrate the sample. The dried peptide sample is reconstituted using a solution comprising 0.1% formic acid (FA) and 3% ACN. This reconstitution is achieved by subjecting the sample to vortexing and ultrasonic agitation in a Bioruptor sonicator for 10 min (30 s on/30 s off, high setting). Throughout this process, the sample is kept in an ice water bath. The prepared samples, equivalent to 1.2 × 10^3^ cells, are subjected to analysis using a quadrupole Orbitrap mass spectrometer (Q-Exactive, Thermo Fisher Scientific) equipped with an EASY-nLC 1000 system (Thermo Fisher Scientific). Tryptic peptides are directly injected into an analytical column and separated using a gradient of solvents A (0.1% FA) and B (90% ACN/0.1% FA) (0–5% B (5 min), 5–32% B (37 min), 32–55% B (15 min), 55–95% B (1 min)). The eluted peptides are subsequently analyzed using a Q Exactive for data-independent acquisition (DIA)-MS, a mass spectrometry technique employed for the quantification and identification of peptides and proteins [25]. MS1 spectra were obtained in the range of 350–770 *m*/*z* at 17,500 resolutions to set an automatic gain control target of 3 × 10^6^ ions and a maximum injection time of 20 ms. MS2 spectra were collected at >200 *m*/*z* at 35,000 resolutions to set an automatic gain control target of 3 × 10^6^ ions, a maximum injection time of “auto”, and collision energy of 27%. The isolation width for MS2 was 8 *m*/*z*, and window patterns of 650–770 *m*/*z* were used. DIA files were evaluated using the DIA-NN software (v.1.6.0) [26] using default parameters, with fixed modifications of carbamidomethyl and variable modifications of Met oxidation.

### 2.5. Correlation between the Proportion of Fb Subsets and OA Pathology

Radiographic knee OA (KOA) progression was assessed by the Kellgren–Lawrence classification [27] and measurement of radiographic JSW [28]. Preoperative pain intensity was evaluated using a VAS for pain (pain: 0 = no pain, 10 = worst possible pain). We performed clinical assessments before each surgical procedure at our outpatient clinic one month before surgery. To investigate the relationship between the proportion of Fb subsets and OA pathology, power analysis was conducted with an alpha of 0.05 and a power of 0.80 in G*POWER3 to determine a suitable sample size. Power analysis revealed that 25 samples were needed to detect a correlation between the VAS score and the proportion of CD39+CD55− cells. Therefore, 25 samples were analyzed to estimate the relationship between the proportion of Fb subsets and OA pathology (JSW, VAS).

### 2.6. Statistical Analysis

Following the Shapiro–Wilk test, Spearman’s correlation coefficient was used to investigate the relationships between VAS and JSW and the proportion of Fb subsets in the synovium of KOA patients.

## 3. Results

### 3.1. Fb Subsets in the Synovium and OA Pathology in KOA Patients

**Figure 1:**
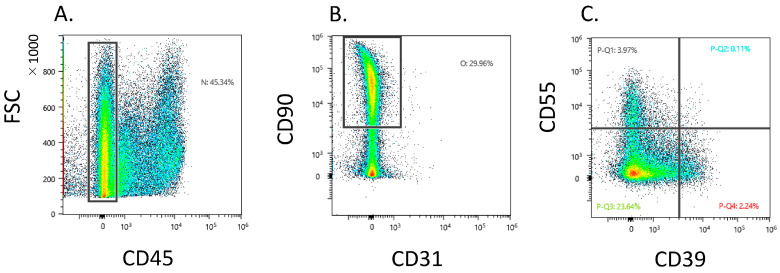
Flow cytometric analysis of CD39+ and CD55+ fibroblast obtained from knee osteoarthritis patients. (**A**) CD45− gate in synovial cells. *X*-axis, forward scattering (FSC); *y*-axis, CD45. (**B**) CD31−CD90+ gate in CD45-negative gate. *X*-axis, CD31; *y*-axis, CD90 (**C**) Dot plot analysis of the fibroblast population. *X*-axis, CD39; *y*-axis, CD55.

Dot plot analysis showed that 37.9 ± 14.5 Fb (CD45−CD31−CD90+) existed in the CD45 negative gate (Figure 1A,B). The CD45−CD31−CD90+ fraction contained a heterogeneous population and proportion of CD39−CD55−, CD39+CD55−, and CD39−CD55+ Fb at 31.2 ± 11.7, 4.1 ± 3.0, and 6.0 ± 4.3, respectively (Figure 1C).

### 3.2. Characterization of CD39+CD55− and CD39−CD55+ Using RNA-Seq and LC/MS

**Figure 2:**
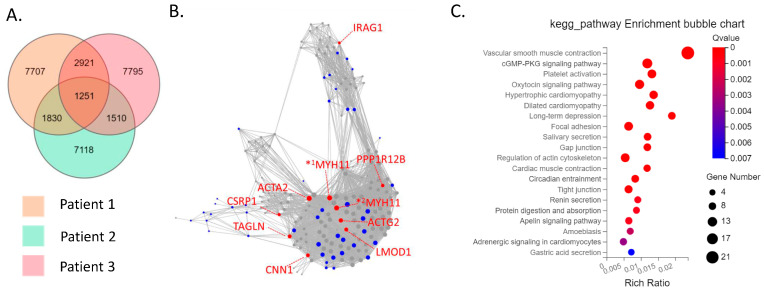
Key driver and pathway analysis of upregulated genes in CD39+CD55− cells. (**A**) Common upregulated genes in CD39+CD55− cells compared to CD39−CD55+ cells among three patients were determined using a Venn diagram. (**B**) Key driver gene analysis of common upregulated genes in CD39+CD55− cells. Blue dots indicate the gene selected to estimate the key driver gene. Red dots indicate key driver genes. *^1^ MYH11 transcript variant SM1A, *^2^ MYH11 transcript variant SM2A (**C**) KEGG analysis of the common upregulated genes in CD39+CD55− cells.

**Figure 3:**
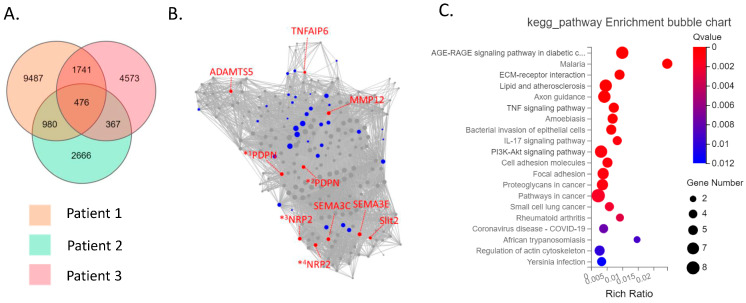
Key driver and pathway analysis of upregulated genes in CD39−CD55+cells. (**A**) Common upregulated genes in CD39−CD55+ cells compared to CD39+CD55− cells among three patients were determined using a Venn diagram. (**B**) Key driver gene analysis of the common upregulated genes in CD39−CD55+ cells. Blue dots indicate the gene selected to estimate the key driver gene. Red dots indicate key driver genes. *^1^ PDPN transcript variant 1, *^2^ PDPN transcript variant 2, *^3^ NRP2 transcript variant 2, *^4^ NRP2 transcript variant 5 (**C**) KEGG analysis of the common upregulated genes in CD39−CD55+ cells.

The CD39−CD55− population contained a higher proportion of cells expressing myeloid cell (CD163, VSIG4, C1QC) markers [29,30,31] than CD39−CD55+ cells (Supplementary Figure S3). Therefore, we analyzed CD39−CD55+ and CD39+CDD55− cells as a fibroblastic population. DEG analysis based on RNA−seq data revealed 1727 genes were significantly differentially expressed between CD39+CD55− and CD39−CD55+ Fb. Among the 1251 upregulated genes (Supplementary Table S1) in CD39+CD55− Fb, 43 genes were selected to estimate key driver genes, and 10 key driver genes were identified in DEGs (Table 1, Figure 2A,B). KEGG pathway analysis of these 10 key driver genes and 43 selected genes was enriched for genes involved in vascular smooth muscle contraction, the Apelin pathway, and cGMP-PKG signaling (Figure 2C). Among the 476 upregulated DEGs in CD39−CD55+ cells (Supplementary Table S3) selected to estimate key driver genes, 10 key driver genes were identified in DEGs (Table 2, Figure 3A,B). KEGG pathway analysis of 10 key driver genes and 34 selected genes was enriched for genes involved in rheumatoid arthritis, the TNF-α signaling pathway, and the IL-17 pathway (Figure 3C).

**Figure 4:**
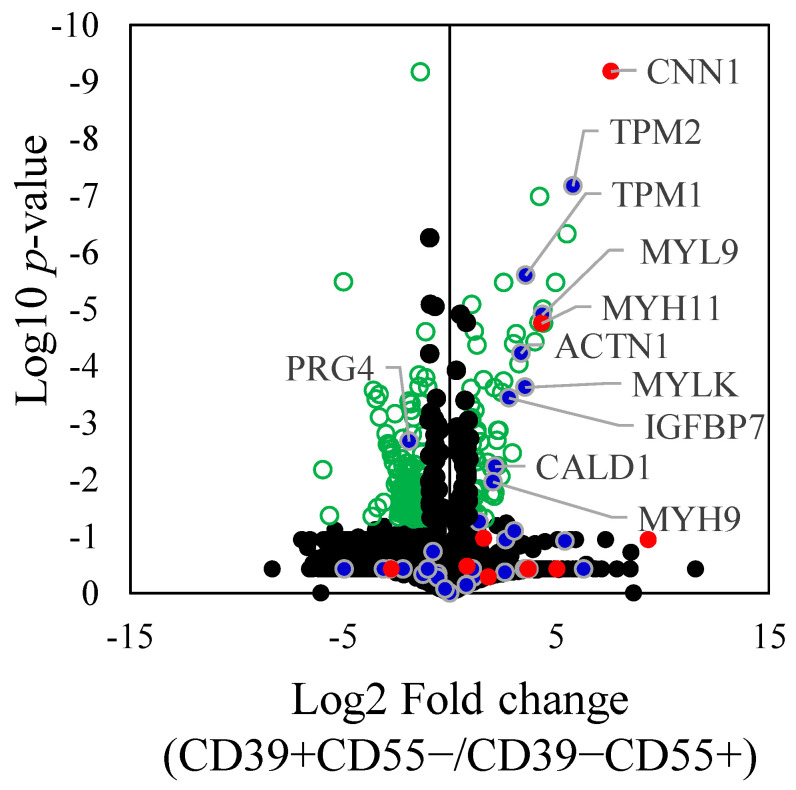
Differently expressed proteins between CD39+CD55− and CD39−CD55+. Differently expressed proteins between CD39+CD55− and CD39−CD55+ were shown using a volcano plot. Green dot. Blue dots indicate the gene selected to estimate the key driver gene. Red dots indicate key driver genes. Statistically significant values (*p* < 0.05) are depicted as green circle, not significant values as black spots.

Proteomic analysis of differentially expressed proteins (DEPs) revealed 193 proteins that were significantly differentially expressed between CD39+CD55− and CD39−CD55+ Fb (Supplementary Table S4). Among the selected and key driver genes in KDA analysis of upregulated genes in CD39+CD55− Fb, 10 proteins encoded by ACTN1, CALD1, CNN1, IGFBP7, MYH11, MYH9, MYL9, MYLK, TPM1, and TPM2 were significantly higher than in CD39−CD55+ Fb (Figure 4). Among the selected and key driver genes in KDA analysis of the upregulated gene in CD39−CD55+ Fb, proteoglycan 4, encoded by PRG4, was significantly higher than that in CD39+CD55−Fb (Figure 4).

### 3.3. Correlation between the Proportion of Fb Subsets and OA Pathology

**Figure 5:**
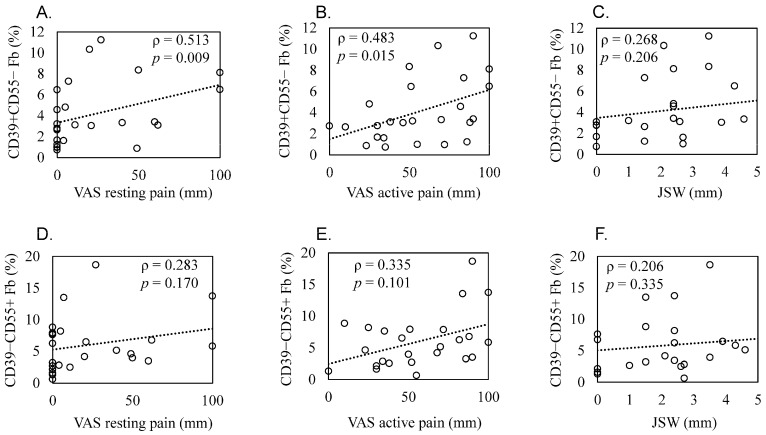
Correlation between the proportion of synovial CD39+CD55− and CD39−CD55+ cells and radiographic joint space width and pain level in knee osteoarthritis patients. Scatter plots showing correlations. Correlation between the proportion of CD39+CD55− fibroblasts and (**A**) resting pain evaluated using a visual analog scale (VAS), (**B**) active pain evaluated using a VAS, and (**C**) radiographic joint space width (JSW). Correlation between the proportion of CD39−CD55+ fibroblasts and (**D**) resting pain evaluated using VAS, (**E**) active pain evaluated using a VAS, and (**F**) JSW. Statistical analysis was performed using Spearman’s correlation coefficient, as indicated by the ρ values. *p* < 0.05 indicates statistical significance.

Patient demographics and clinical features are shown in Table 3. Scatterplots of these correlations are shown in Figure 5A–F. The proportion of CD39+CD55− Fb in synovium significantly correlated with resting and active pain levels in KOA patients (resting pain, ρ = 0.513, *p* = 0.009; active pain, ρ = 0.483, *p* = 0.015, Figure 5A,B). There was no correlation between JSW and the proportion of CD39+CD55− Fb (ρ = 0.268, *p* = 0.206, Figure 5C). In contrast, we saw no correlation between the proportion of CD39−CD55+ Fb and resting pain (ρ = 0.283, *p* = 0.170, Figure 5D), active pain (ρ = 0.335, *p* = 0.101, Figure 5E), and JSW (ρ = 0.206, *p* = 0.335, Figure 5F).

## 4. Discussion

Myogenic trans differentiation of fibroblasts to smooth muscle-like cells (SMC) showed increased myogenic markers, including CNN1, MYH11, IGFPBP7, and TPM1 [32,33,34,35,36]. Notably, our study observed higher expression of these myogenic markers in synovial CD39+CD55− fibroblasts compared to CD39−CD55+ fibroblasts. In contrast, several markers overlapped between myofibroblast and smooth muscle cells. A previous study reported that myofibroblasts selectively express Postn-encoding periostin and that the deletion of periostin (+) myofibroblasts reduces collagen production and scar formation after myocardial infraction in mice [35]. The Fb subset expressing POSTN was identified in RA synovium and highly expressed collagen genes [37]. In our study, the POSTN gene was selected to estimate key driver genes in CD39+CD55−. Our results suggested that the CD39+CD55− population exhibits a myofibroblast-like phenotype.

Synovial fibrosis factor is responsible for causing joint pain and stiffness in arthritis [38]. A prior study proposed that when synovial fibroblasts are exposed to stiffer environments due to fibrosis, their characteristics deviate from their essential role in producing lubricating substances [39]. It is well established that lubricin, encoded by the PRG4 gene, plays a crucial role in regulating cartilage health [40,41]. Since synovial fibroblasts are considered a primary source of lubricin [42,43], a reduced expression of lubricin in the context of fibrosis could potentially contribute to the development of osteoarthritis. Our findings showing reduced expression of PRG4 in the CD39+CD55− population, which correlates with pain scores in KOA patients, support the idea that altered PRG4 expression may contribute to osteoarthritic pain. However, the direct relationship between PRG4 expression in fibroblasts and its impact on OA progression and pain requires further investigation. Our study proposes a potential therapeutic avenue for managing osteoarthritic pain by targeting the CD39+CD55− cell population. This suggestion aligns with recent studies that have explored a small-molecule inhibitor targeting ROCK to mitigate the transition from fibroblasts to myofibroblasts in diseased synovial tissue [39]. Decreasing the CD39+CD55− cell population may indeed hold promise, but further research is necessary to validate this approach.

The pain-related pathways identified in the study, including the Apelin pathway and cGMP-PKC signaling pathway, are intriguing. The association of synovial apelin expression with OA pain and the modulation of the cGMP signaling pathway to attenuate pain in OA patients [34,35,36] further support the notion that these pathways could contribute to the pain experienced by KOA patients. This finding opens avenues for investigating targeted interventions to alleviate pain.

The presence of PRG4+ fibroblasts and their association with disease activity in osteoarthritis and rheumatoid arthritis [7,37] underscores the importance of understanding the roles of specific fibroblast subtypes in disease progression. Prg4+ synovial fibroblasts expressed Rspo2, which may contribute to pathological crosstalk between joints in the post-traumatic osteoarthritis model [7,37]. Recent analysis of single cells in the synovium of RA patients has also shown that the presence of PRG4+ fibroblasts is associated with the level of disease activity, indicating their potential involvement in the progression of RA [37]. CD39−CD55+ Fb highly expressed PRG4 mRNA and protein compared to CD39+CD55 Fb. In addition, CD39−CD55+ Fb highly expressed Rspo2 and inflammatory genes, suggesting that CD39−CD55+Fb has an inflammatory phenotype in synovium.

While our study did not establish a direct association between the proportion of CD39−CD55+ cells and OA pathology or pain scores, it is essential to consider the dynamic nature of fibroblast subtypes across various stages of OA progression. Previous research has shown that fibroblast phenotypes are associated with OA progression and pain [8], and synovial tissues from early-stage OA, particularly from painful sites, exhibit distinct gene expression profiles, with upregulation of genes like CXCL1 and INHBA, which are also upregulated in CD39−CD55+ cells. Furthermore, IGFBP7, downregulated during OA progression [8], displayed lower expression in CD39−CD55+ cells compared to CD39+CD55−. These findings underscore the complexity of fibroblast involvement in OA pathogenesis. Importantly, the contribution of CD39−CD55+ cells may be more significant in the early stages of OA, where synovitis plays a crucial role in driving pain [44]. As OA advances, the inflammatory response tends to diminish [45,46], potentially diminishing the impact of this fibroblast subset in late-stage OA. To fully elucidate the role of fibroblast subtypes across different OA stages, further investigations utilizing synovial tissue from early OA patients are warranted.

It is crucial to acknowledge the limitations of our study. First, we did not include a comparison with healthy individuals, which would provide essential context for understanding the differences observed in OA patients. Second, we did not evaluate inflammation in synovial samples. Additionally, the localization of fibroblast subsets and their direct relationship with pain remain areas that need further investigation. Addressing these limitations will contribute to a more comprehensive understanding of the role of synovial fibroblasts in OA.

In conclusion, our study sheds light on the potential significance of CD39+CD55− synovial fibroblasts in osteoarthritis, their myofibroblast-like phenotype, and their association with joint pain. These findings provide a foundation for further research into the mechanisms underlying fibrosis, the impact of altered gene expression on osteoarthritic joints, and potential therapeutic strategies.

## Figures and Tables

**Table 1:** Key driver gene analysis of 1251 upregulated genes in CD39+CD55− fibroblasts.

NCBI RNA ID	Symbol	log2	*p* Value	NCBI RNA ID	Symbol	log2	*p* Value
**NM_001613**	**ACTA2**	4.33	3.1 × 10^−5^	**NM_002474**	MYH11 *^14^	4.74	1.6 × 10^−3^
**NM_001615**	**ACTG2**	6.16	5.9 × 10^−4^	**NM_022844**	MYH11 *^15^	9.56	8.1 × 10^−7^
NM_001130005	ACTN1 *^1^	3.78	2.0 × 10^−2^	NM_002473	MYH9	1.53	1.9 × 10^−1^
NM_001102	ACTN1 *^2^	1.58	2.1 × 10^−1^	NM_079423	MYL6	1.56	1.6 × 10^−1^
NM_004342	CALD1 *^3^	2.73	4.6 × 10^−3^	NM_006097	MYL9	2.76	6.0 × 10^−3^
NM_033138	CALD1 *^4^	2.62	2.6 × 10^−2^	NM_053032	MYLK *^16^	5.61	1.4 × 10^−3^
**NM_001299**	**CNN1**	5.59	2.9 × 10^−3^	NM_053031	MYLK *^17^	5.73	7.5 × 10^−3^
XM_005273081	CNST	0.89	4.5 × 10^−1^	NM_002508	NID1	1.68	7.5 × 10^−2^
NM_001855	COL15A1	2.35	4.2 × 10^−2^	NM_001204376	NPR3	1.53	1.5 × 10^−1^
NM_030582	COL18A1	2.80	7.9 × 10^−2^	NM_033430	PDE5A	5.17	2.5 × 10^−4^
NM_001845	COL4A1 *^5^	2.26	4.8 × 10^−2^	NM_001135936	POSTN *^18^	1.90	2.1 × 10^−1^
NM_001303110	COL4A1 *^6^	2.14	4.1 × 10^−2^	NM_001286667	POSTN *^19^	2.88	1.2 × 10^−1^
NM_001846	COL4A2	2.20	8.3 × 10^−2^	**NM_001197131**	**PPP1R12B**	2.85	5.6 × 10^−2^
**NM_004078**	**CSRP1**	1.43	1.5 × 10^−1^	NM_001098512	PRKG1 *^20^	2.65	6.6 × 10^−3^
NM_004006	DMD *^7^	3.76	1.0 × 10^−1^	NM_006258	PRKG1 *^21^	1.07	4.4 × 10^−1^
NM_004015	DMD *^8^	3.54	1.8 × 10^−2^	NM_001321643	ROCK2	0.89	4.7 × 10^−1^
NM_004016	DMD *^9^	4.30	2.6 × 10^−3^	NM_000602	SERPINE1	3.61	2.3 × 10^−2^
NM_001130684	GUCY1A1	9.55	7.7 × 10^−7^	NM_003118	SPARC	0.89	3.5 × 10^−1^
NM_000855	GUCY1A2	2.27	2.7 × 10^−2^	NM_001001522	TAGLN *^22^	2.15	1.5 × 10^−1^
NM_000857	GUCY1B1	2.83	1.3 × 10^−3^	**NM_003186**	TAGLN *^23^	3.20	7.8 × 10^−3^
NM_014571	HEYL	3.96	4.6 × 10^−3^	NM_001018004	TPM1 *^24^	3.64	4.3 × 10^−2^
NM_001553	IGFBP7 *^10^	2.37	4.8 × 10^−3^	NM_001018007	TPM1 *^25^	3.27	6.3 × 10^−3^
NM_001253835	IGFBP7 *^11^	2.30	2.0 × 10^−1^	NM_001018020	TPM1 *^26^	9.78	2.8 × 10^−7^
**NM_001100163**	**IRAG1**	5.44	1.5 × 10^−3^	NM_213674	TPM2	3.76	8.4 × 10^−5^
NM_002222	ITPR1 *^12^	2.02	3.9 × 10^−2^	NM_001043351	TPM3 *^27^	2.30	2.8 × 10^−1^
NM_001099952	ITPR1 *^13^	1.64	1.2 × 10^−1^	NM_001278188	TPM3 *^28^	2.75	1.1 × 10^−1^
**NM_012134**	**LMOD1**	4.03	1.2 × 10^−3^				

Normal letters indicate the gene selected to estimate the key driver gene. Bold letters indicate key driver genes. *^1^ ACTN1 transcript variant 2, *^2^ ACTN1 transcript variant 3, *^3^ CALD1 transcript variant 2, *^4^ CALD1 transcript variant 1, *^5^ COL4A1 transcript variant 2, *^6^ COL4A1 transcript variant 1, *^7^ DMD transcript variant Dp427m, *^8^ DMD transcript variant Dp71, *^9^ DMD transcript variant Dp71b, *^10^ IGFBP7 transcript variant 2, *^11^ IGFBP7 transcript variant 1, *^12^ ITPR1 transcript variant 1, *^13^ ITPR1 transcript variant 2, *^14^ MYH11 transcript variant SM1A, *^15^ MYH11 transcript variant SM2A, *^16^ MYLK transcript variant 8, *^17^ MYLK transcript variant 7, *^18^ POSTN transcript variant 4, *^19^ POSTN transcript variant 7, *^20^ PRKG1 transcript variant 1, *^21^ PRKG1 transcript variant 2, *^22^ TAGLN transcript variant 1, *^23^ TAGLN transcript variant 2, *^24^ TPM1 transcript variant Tpm1.6, *^25^ TPM1 transcript variant Tpm1.4, *^26^ TPM1 transcript variant Tpm1.3, *^27^ TPM3 transcript variant Tpm3.2, *^28^ TPM3 transcript variant 6.

**Table 2:** Key driver gene analysis of 476 upregulated genes in CD39−CD55+ fibroblasts.

RNA ID	Symbol	log2	*p* Value	RNA ID	Symbol	log2	*p* Value
NM_007038	**ADAMTS5**	3.26	4.84 × 10^−4^	NM_002422	MMP3	3.74	1.00 × 10^−2^
NM_052866	ADAMTSL1	3.14	1.56 × 10^−2^	NM_003872	NRP2 *^7^	1.68	1.50 × 10^−1^
XM_006718223	CD82	2.82	4.82 × 10^−2^	NM_201267	NRP2 *^8^	1.53	8.30 × 10^−2^
NM_001293304	CEMIP	2.98	1.25 × 10^−1^	NM_002593	PCOLCE	1.86	1.09 × 10^−1^
NM_016929	CLIC5	2.98	9.98 × 10^−2^	NM_006474	PDPN *^9^	2.50	1.87 × 10^−1^
XM_006713500	CP	2.52	2.55 × 10^−1^	NM_198389	PDPN *^10^	2.36	4.50 × 10^−2^
NM_001511	CXCL1	2.98	8.95 × 10^−4^	NM_001127708	PRG4 *^11^	4.85	1.93 × 10^−4^
NM_001291807	FAP	9.82	2.04 × 10^−5^	NM_001127709	PRG4 *^12^	3.05	3.12 × 10^−3^
NM_004464	FGF5	3.72	3.88 × 10^−2^	NM_001127710	PRG4 *^13^	2.94	4.36 × 10^−3^
NM_002026	FN1 *^1^	2.84	2.74 × 10^−3^	NM_002999	SDC4	2.47	2.36 × 10^−1^
NM_054034	FN1 *^2^	4.22	2.84 × 10^−2^	NM_003005	SELP	2.53	1.08 × 10^−1^
NM_212474	FN1 *^3^	3.49	3.28 × 10^−4^	XM_005245440	SELP *^14^	4.22	1.56 × 10^−3^
NM_212476	FN1 *^4^	3.46	3.98 × 10^−4^	NM_006080	SEMA3A	3.75	8.78 × 10^−4^
NM_005708	GPC6	2.27	2.25 × 10^−2^	XM_005250110	SEMA3A *^15^	11.07	1.64 × 10^−6^
NM_002775	HTRA1	3.01	6.57 × 10^−3^	XM_006715839	SEMA3A *^16^	9.96	1.58 × 10^−7^
NM_002192	INHBA	2.85	1.14 × 10^−2^	**NM_006379**	**SEMA3C**	**3.02**	**4.81 × 10^−3^**
NM_001166449	ITIH4 *^5^	2.51	1.58 × 10^−2^	**NM_012431**	**SEMA3E**	**3.33**	**3.65 × 10^−3^**
NM_002218	ITIH4 *^6^	3.03	4.00 × 10^−2^	**NM_004787**	**SLIT2**	**2.68**	**7.93 × 10^−2^**
XM_006715990	MET	2.13	2.15 × 10^−1^	**NM_007115**	**TNFAIP6**	**2.64**	**2.79 × 10^−2^**
NM_002421	MMP1	3.14	1.06 × 10^−2^	NM_001078	VCAM1 *^17^	1.97	3.47 × 10^−2^
NM_002426	MMP12	3.51	2.91 × 10^−2^	NM_080682	VCAM1 *^18^	2.92	2.58 × 10^−2^
NM_002427	MMP13	5.42	2.63 × 10^−5^	NM_005429	VEGFC	2.73	2.00 × 10^−2^

Normal letters indicate the gene selected to estimate the key driver gene. Bold letters indicate key driver gene. *^1^ FN transcript variant 3, *^2^ FN transcript variant 7, *^3^ FN transcript variant 6, *^4^ FN transcript variant 5, *^5^ ITIH4 transcript variant 2, *6 ITIH4 transcript variant 1, *^7^ NRP2 transcript variant 2, *^8^ NRP2 transcript variant 5, *^9^ PDPN transcript variant 1, *^10^ PDPN transcript variant 2, *^11^ PRG4 transcript variant B, *^12^ PRG4 transcript variant C, *^13^ PRG4 transcript variant D, *^14^ SELP transcript variant X5, *^15^ SEMA3A transcript variant X1, *^16^ SEMA3A transcript variant X3, *^17^ VCAM1 transcript variant 1, *^18^ VCAM1 transcript variant 2.

**Table 3:** Clinical characteristics of knee osteoarthritis patients.

Age (years)	74.5 ± 8.6
Sex, male/female, N	6/19
BMI (kg/m^2^)	29.5 ± 7.4
KL grade (3/4), N	6/19
JSW (mm)	2.4 ± 1.9
VAS resting pain (mm)	22.2 ± 31.2
VAS at active pain (mm)	56.4 ± 29.0

All data are reported as mean ± SD. Abbreviations: BMI, body mass index; JSW, joint space width; VAS, visual analog scale.

## Data Availability

The entire set of RNA-Seq raw data has been submitted to the DNA Data Bank of Japan (DDBJ) and has been assigned the accession code DRA017287. All LC-MS/MS raw data files were deposited in the ProteomeXchange Consortium (http://proteomecentral.proteomexchange.org, accessed on 2 August 2023) via the jPOST partner repository (http://jpostdb.org, accessed on 2 August 2023) 42 as identifiers PXD044350 for ProteomeXchange and JPST002269 for jPOST. The data that support the findings of this study are available on request from the corresponding author, K.U.

## Supplementary Materials

The following supporting information can be downloaded at: https://www.mdpi.com/article/10.3390/biomedicines11113047/s1, Figure S1: Flow cytometric analysis of synovial samples obtained from knee osteoarthritis patients; Figure S2: Flow cytometric analysis of synovial samples obtained from knee osteoarthritis patients; Figure S3: Key driver and pathway analysis of upregulated genes in CD39−CD55− cells; Table S1: Antibodies used in this study; Table S2: Upregulated DEGs in CD39+CD55− cells compare to CD39−CD55+; Table S3: Upregulated DEGs in CD39−CD55+ cells compare to CD39+CD55−; Table S4: Differentially expressed proteins (DEPs) between CD39+CD55− and CD39−CD55+ cells.
