# Supplementary material for: CD39+CD55− Fb Subset Exhibits Myofibroblast-Like Phenotype and Is Associated with Pain in Osteoarthritis of the Knee
Source: Biomedicines. 2023 Nov 14;11(11):3047. doi: 10.3390/biomedicines11113047 (PMC10669511; doi:10.3390/biomedicines11113047)
Supplement: Supplementary file 1 [file biomedicines-11-03047-s001.zip › Supplementary Figure S1.pdf]

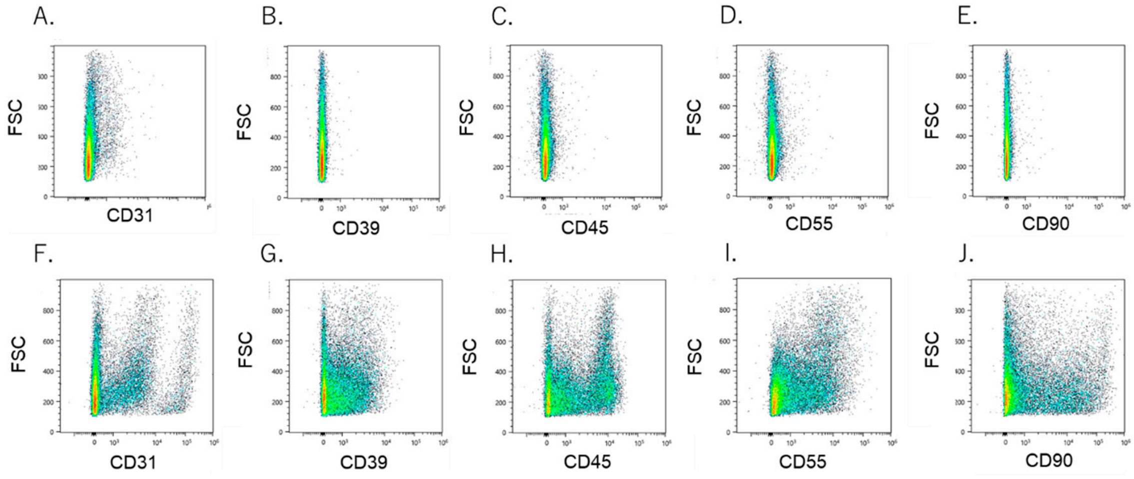

Supplementary Figure S1. Flow cytometric analysis of synovial samples obtained from knee osteoarthritis patients. (A-E) No staining control for (A) CD31, (B) CD39, (C) CD45, (D) CD55, and (E) CD90. (F-J) Single-stained samples for (F) CD31, (G) CD39, (H) CD45. (I) CD55, and (J) CD90.
