# Supplementary material for: CD39+CD55− Fb Subset Exhibits Myofibroblast-Like Phenotype and Is Associated with Pain in Osteoarthritis of the Knee
Source: Biomedicines. 2023 Nov 14;11(11):3047. doi: 10.3390/biomedicines11113047 (PMC10669511; doi:10.3390/biomedicines11113047)
Supplement: Supplementary file 1 [file biomedicines-11-03047-s001.zip › Supplementary Figure S2.pdf]

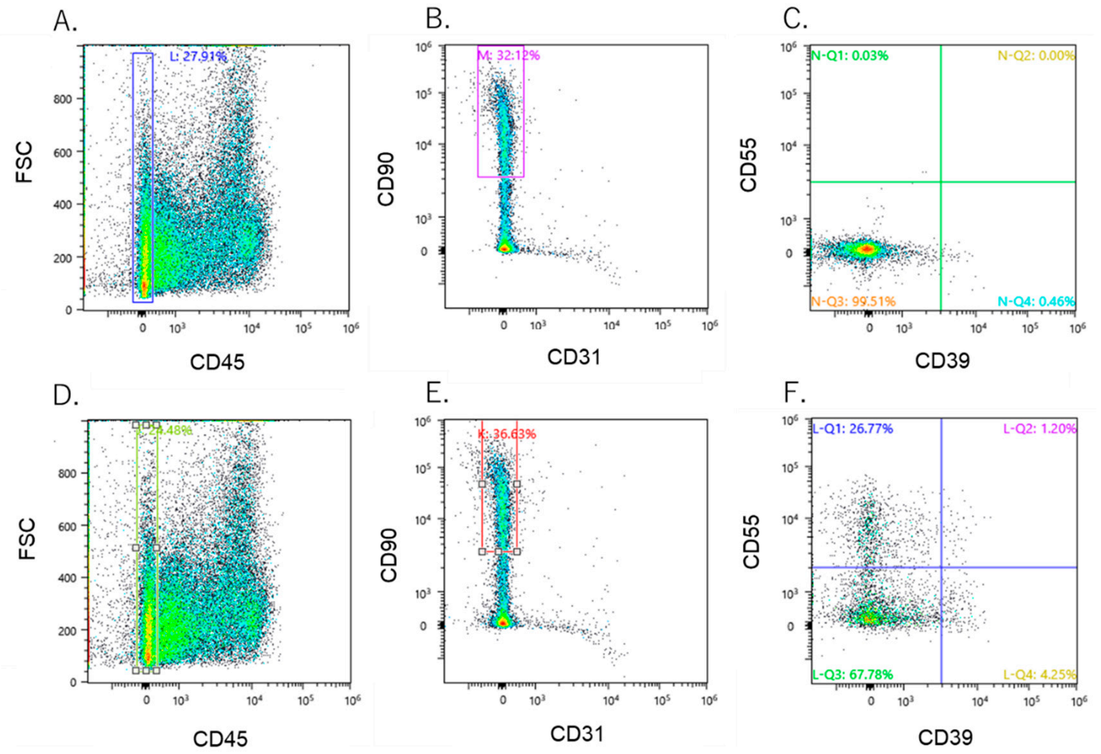

Supplementary Figure S2. Flow cytometric analysis of synovial samples obtained from knee osteoarthritis patients. (A-C) Synovial cells that were treated with anti-CD31, CD45, and CD90 antibodies but not with an-ti-CD39 and CD55 antibodies. (D-F) Synovial cells that were treated with anti-CD31, CD39, CD45, CD55, and CD90 antibodies.
