# Supplementary material for: CD39+CD55− Fb Subset Exhibits Myofibroblast-Like Phenotype and Is Associated with Pain in Osteoarthritis of the Knee
Source: Biomedicines. 2023 Nov 14;11(11):3047. doi: 10.3390/biomedicines11113047 (PMC10669511; doi:10.3390/biomedicines11113047)
Supplement: Supplementary file 1 [file biomedicines-11-03047-s001.zip › Supplementary Figure S3.pdf]

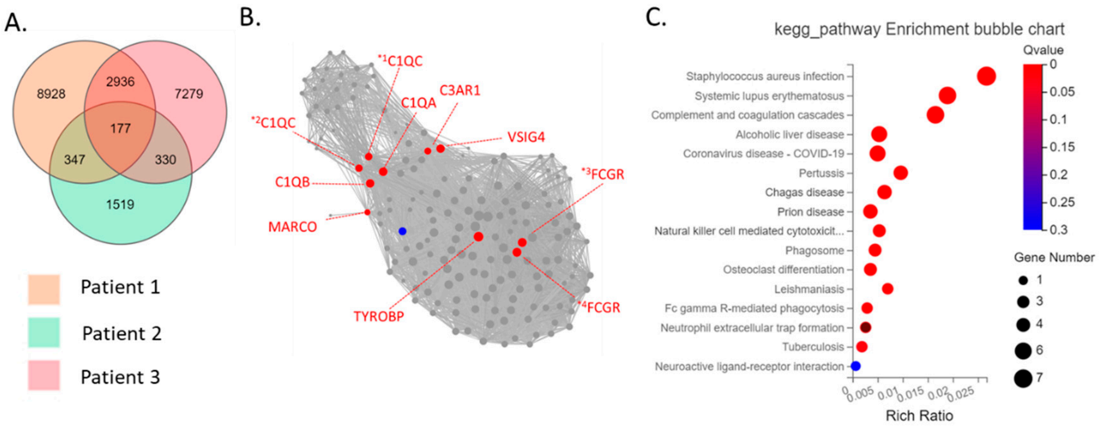

Supplementary Figure S3. Key driver and pathway analysis of upregulated genes in CD39-CD55- cells. (A) Common up-regulated genes in CD39-CD55- cells compared to CD39-CD55+ cells among three patients were determined using a Venn diagram. (B) Key driver gene analysis of common upregulated genes in CD39-CD55- cells. (C) KEGG analysis of the common upregulated genes in CD39-CD55- cells.
