# Supplementary material for: CD39+CD55− Fb Subset Exhibits Myofibroblast-Like Phenotype and Is Associated with Pain in Osteoarthritis of the Knee
Source: Biomedicines. 2023 Nov 14;11(11):3047. doi: 10.3390/biomedicines11113047 (PMC10669511; doi:10.3390/biomedicines11113047)
Supplement: Supplementary file 1 [file biomedicines-11-03047-s001.zip › Supplementary Table S1.pdf]

Supplementary Table S1. Antibodies used in this study

Antibody	isotype	conjugate	Clone	Catalog No.
CD31	Mouse IgG1, κ	Brilliant violet 421	WM59	303124
CD39	Mouse IgG1, κ	APC/Cyanine7	A1	328226
CD45	Mouse IgG1, κ	FITC	HI30	304006
CD55	Mouse IgG1, κ	PE	JS11	311308
CD90	Mouse IgG1, κ	PE/Cyanine7	5E10	328124

All antibodies purchased from Biolegend (San Diego, CA, USA).
